# RAS/RAF mutations in tumor samples and cell-free DNA from plasma and bone marrow aspirates in multiple myeloma patients

**DOI:** 10.7150/jca.43729

**Published:** 2020-03-15

**Authors:** Qian Li, Helen J Huang, Jing Ma, Yafei Wang, Zeng Cao, George Karlin-Neumann, Filip Janku, Zhiqiang Liu

**Affiliations:** 1 Tianjin Medical University Cancer Institute and Hospital; National Clinical Research Center for Cancer; Tianjin Key Laboratory of Cancer Prevention and Therapy; Tianjin's Clinical Research Center for Cancer, Tianjin, China; 2 Department of Investigational Cancer Therapeutics (Phase I Clinical Trials Program), the University of Texas MD Anderson Cancer Center, Houston, Texas, USA; 3 Bio-Rad Laboratories, Pleasanton, California, USA; 4 Tianjin Key Laboratory of Cellular Homeostasis and Human Diseases, School of Basic Medical Science, Tianjin Medical University; Department of Physiology and Pathophysiology, School of Basic Medical Science, Tianjin Medical University, Heping, Tianjin, 300070, China

**Keywords:** multiple myeloma, RAS mutations, ddPCR

## Abstract

**Purpose**: To evaluate the detection of gene mutations in bone marrow biopsy and circulating free DNA (cfDNA) from plasma in multiple myeloma (MM).

**Experimental design**: We used cell-free DNA from plasma and bone marrow to test *BRAF V600*, *KRAS G12/G13*, *NRAS G12/G13* and *NRAS Q61* mutations using multiplex assays for droplet digital PCR (ddPCR), and evaluated results with clinical outcomes.

**Results**: We found of 83 patients, the detectable mutation frequencies for the above four genes were 4 (5%), 13 (16%), 3 (4%) and 14 (17%) in bone marrow, respectively. The median variant allelic frequency (VAF) in most mutations were 1.595%. In 17 paired cfDNA samples, the detectable mutation frequencies for the above four genes were 5 (30%), 1 (6%), 0 (0%) and 3 (18%) respectively, and the median VAF rate was 2.9%. Agreement between bone marrow DNA and plasma cfDNA were 76%, 100%, 100% and 100% compared to the tissue detections, respectively. In 17 patients with paired bone marrow and plasma samples, the above four mutations were 3 (18%), 1 (6%), 0 (0%) and 2 (12%) respectively, with the agreement rates of 88%, 88%, 100% and 100% compared to tissue detections. Of 57 patients with available outcome data, high mutation VAF had a shorter median survival than patients with low mutation VAF (*P*=0.0322).

**Conclusions**: Oncogenic mutations in *BRAF*, *KRAS* and *NRAS* genes can be detected in the bone marrow and plasma cfDNA with ddPCR in patients with MM patients and high VAF is associated with short survival.

## Introduction

Multiple myeloma (MM) is a genetically highly heterogeneous disease 1. Despite the successful applications of novel agents, such as immunomodulatory drugs and proteasome inhibitors, and improvement in response and survival rates, MM patients eventually undergo drug resistance and refractory 2, 3. In addition to the recurrent disclosed cytogenetic and molecular abnormalities including translocations of the immunoglobulin heavy chain locus, chromosomal trisomies, partial deletions or monosomies of chromosomes 13, 1p and 17p, somatic mutations in *RAS/RAF* genes are also highly considered as the driver factors for MM tumorigenesis and drug resistance 4, 5. *KRAS*, *NRAS* and *BRAF* mutations are detectable in up to 50% of newly diagnosed MM patients 6, 7. Furthermore, some preclinical and early clinical studies suggest that *RAS/RAF* mutations may predict poor prognosis of MM 8. These genes encode proteins with a key role in the mitogen- activated protein kinase pathway and therefore are considered to be major therapeutic targets in MM as in many other cancers 9, 10. Therefore, detection of *RAS* mutations becomes more and more important in the clinic with the development of personalized therapy.

The current method for isolation of tumor DNA from MM patients requires collection of bone marrow aspirates which is invasive, often painful, and associated with significant economic cost. To enrich the malignant plasma cells, bone marrow aspirates often need to be isolated by flow cytometry or antibody-coated magnetic microbeads. In addition, MM consists of multiple subclones and the tumor cells may infiltrate throughout the bone marrow by the way of multi-focal deposits 11. Thus, molecular testing from a single bone marrow biopsy site may inadequately represent the complete tumor genetic diversity.

Previous studies have demonstrated that cell- free DNA (cfDNA) from plasma, which is sloughed or secreted into the circulation by tumor cells and cells in the tumor microenvironment, may contain a more complete representation of the entire tumor genome 12, 13. Hybrid-capture and next-generation- sequencing (NGS), which targets mutation hotspots such as *RAS* mutations using cfDNA, has become popular in solid tumors 12. Recently, monitoring of somatic mutations in plasma has been demonstrated in MM using droplet digital PCR (ddPCR) 14. ddPCR is based on partitioning of a PCR sample into tens of thousands of uniformly sized droplets in oil which are thermocycled to endpoint, then individually scored for fluorescence of the desired targets enabling direct quantification of each of these. This technology does not need calibration curves and has better inherent sensitivity and specificity than standard quantitative PCR or NGS, and has a simpler workflow than other digital PCRs such as BEAMing 15, 16.

In the current study, we evaluate the detection and quantification of common oncogenic mutations in *BRAF*, *KRAS* and *NRAS* genes in non-pre-amplified plasma cfDNA by multiplexed ddPCR. We further assess whether this method has acceptable concordance with tumor DNA testing, as well as study the relationship between the level of these mutant alleles in plasma and the clinical outcome of MM patients.

## Methods

### Patients

This study enrolled 83 diagnosed MM patients from Hematology and Blood Marrow Transplantation department of Tianjin Medical University Cancer Institute and Hospital from 2010 to 2016. All patients diagnosed with multiple myeloma according to the criteria made by WHO in 2015 were included, but those had other kinds of tumors were excluded. Among all patients 46 were treated and the others were not treated by any chemotherapy regimen. Bone marrow aspirates were obtained from all 83 patients and 17 peripheral blood (PB) samples were provided simultaneously. All patients provided written informed consent to the sampling and clinical data collection. This study was approved by the institutional ethics committee of Tianjin Medical University Cancer Institute and Hospital.

### Isolation of bone marrow mononuclear cells, determination of MM cell proportion and isolation of CD138^+^ plasma cells

Briefly, active MM patients with ≥30% tumor burden were subject to Ficoll isolation of bone marrow mononuclear cells; for those with tumor burden <30%, bone marrow mononuclear cells were enriched by CD138^+^ antibody-coated magnetic microbeads (Miltenyi Biotec, Bergisch Gladbach, Germany). DNA from isolated tumor cells was extracted by using the QIAamp DNA Mini Kit (QIAGEN GmbH, Hilden, Germany).

### Peripheral blood collection and processing

Peripheral blood samples (10 ml) were obtained simultaneously with collection of bone marrow samples. Briefly, plasma samples were centrifuged at 820×g for 10 min, supernatant was collected without disturbing the cellular layer and centrifuged again at 16,000×g for 10 min to remove any residual cellular debris and stored at -80ºC in 1 ml aliquots for long-term storage until isolation of cfDNA.

### Cell-free DNA extraction

Frozen plasma samples were used for cfDNA extraction using the QIAamp Circulating Nucleic Acid kit (Qiagen, Valencia, CA). An average of 3 ml of plasma was used for cfDNA extractions. Subsequently, cfDNA was quantified with a QUBIT Fluorometer 2.0 and high sensitivity DNA detection kits (Life Technology, Carlsbad, CA, USA). The extracted DNA was stored at -80℃ until further processing.

### *BRAF*, *KRAS* and *NRAS* gene mutation Testing

From each tumor DNA or cfDNA sample, a total of 16 ng unamplified DNA was tested after integrity evaluated by OD260/280 (NanoDrop 3000, ThermoFisher, Wilmington, MA, USA) with each of the following multiplex ddPCR Screening Kits (Bio-Rad, Pleasanton, CA): *BRAF* V600Mx (V600E, V600K, V600R); *KRAS* Mx (G12A, G12C, G12D, G12S, G12V, G13D, G13R, G13V); *NRAS G12/G13, NRAS Q61* according to the procedure described in our previous report 17. ddPCR was performed by using the QX200 Droplet Digital PCR platform (Bio-Rad, Hercules, CA, USA) according to the manufacturer's standard protocol. Each ddPCR sample reaction was partitioned into 20,000 droplets followed by thermocycling, where endpoint amplification of the template molecules occurs in each individual droplet. The investigators performing the mutation analysis of the cfDNA samples were blinded to the results of the tumor DNA samples and used appropriate positive and negative controls.

### Statistical analysis

Clinical data were collected retrospectively from internal digital and external document records. All statistical analyses were performed using GraphPad Prism 7.0. Overall survival (OS) was defined as the time from the date of study entry to the date of death or last follow-up. The Kaplan-Meier method was used to estimate OS, and a log-rank test was used to compare OS among patient subgroups. Cox proportional hazards regression models were fit to assess the association between patient characteristics. All tests were two-sided, and *P* values less than 0.05 were considered statistically significant. Detailed descriptions are provided within the results and figure legends.

## Results

### Patient characteristics

83 newly diagnosed MM patients were diagnosed and classified according to the criteria of International Myeloma Working Group. The patients' median age was 63 years and most of them were older than 60 years (N=55, 66%). Most patients were male (N=54, 65%; male: female=1.86:1), and 40% (N=33) of them were at the stage III according to the International Staging System (ISS). The most common immunotyping was IgG (N=34, 41%). 57 patients have complete follow-up data and the median follow-up time was 24.5 months (range, 0.2-89 months). In total, 46 patients were administered with at least one cycle of anti-myeloma treatment and 37 of them were treated with novel agents-based therapy regimens, including bortezomib, thalidomide or lenalidomide. Detailed patients' characteristics are listed in **Table 1**.

### *BRAF*, *KRAS* and *NRAS* mutations in bone marrow tumor DNA and plasma cfDNA

In our cohort, 28 of 83 (34%) patients had *BRAF V600Mx*, *KRAS Mx*, *NRAS G12/G13* and/or *NRAS Q61* mutations in their bone marrow tumor DNA sample (4 [5%] *BRAF V600Mx* mutations, 13 [16%] *KRAS Mx* mutations, 3 [4%] *NRAS G12/G13* mutations, 14 [17%] *NRAS Q61* mutations). Of all the 28 patients with mutations, 24 (86%) had single mutation, 2 (7%) had two mutations and 2 (7%) had three simultaneous mutations (**Table 2**).

Coincident with collection of bone marrow samples, 17 patients also donated blood samples which were tested for tumor mutations in plasma cfDNA. Among the 17 patients, 9 (53%) had *BRAF* or *RAS* mutations of which 5 [30%] had a *BRAF V600Mx* mutation (**Table 2**). It is noteworthy that concordance between tumor and plasma samples was 100% for all mutations with the exception of BRAF, where 4 additional plasma samples had detectable mutations not seen in their matched tumor samples (Table 3). Thus, the concordance of cfDNA and tumor DNA for *BRAF V600Mx* was 76%. Only a single plasma sample had a *KRAS Mx* mutation which was also seen in the tumor; the *NRAS Q61* mutation was seen in 3 cases, both in cfDNA and tumor DNA; and no mutations in *NRAS G12/G13* were detected in any of these 17 cfDNA samples or their matched tumors (**Table 3**).

### *BRAF*, *KRAS* and *NRAS* mutations and survival

**Figure 1:**
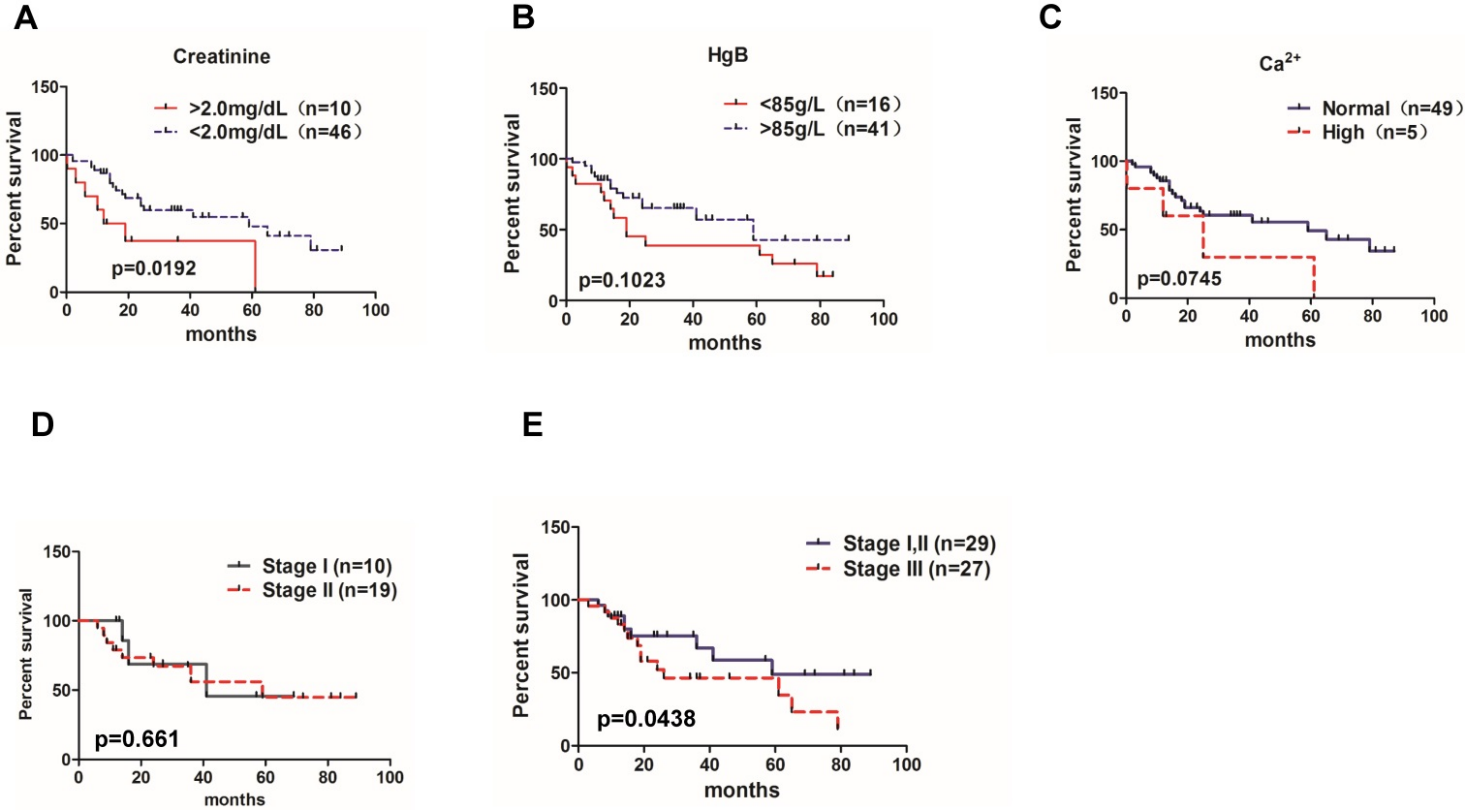
** Significance of MM prognostic biomarkers on the patient's overall survival.** Correlations of the overall survival rate of all 83 MM patients with **(A)** creatinine level (95% CI of rate, -0.1601 to 0.6856), **(B)** HgB (95% CI of rate, -0.1480 to 0.7921), and **(C)** Ca^2+^ concentration(95% CI of rate, 2.017 to 2.703). **(D)** Overall survival rate between MM patients in ISS stage I and stage II (95% CI of rate, 0.1721 to 0.9753). **(E)** Overall survival rate between MM patients in ISS stage I,II and stage III (95% CI of rate, 0.2645 to 4.640).

**Figure 2:**
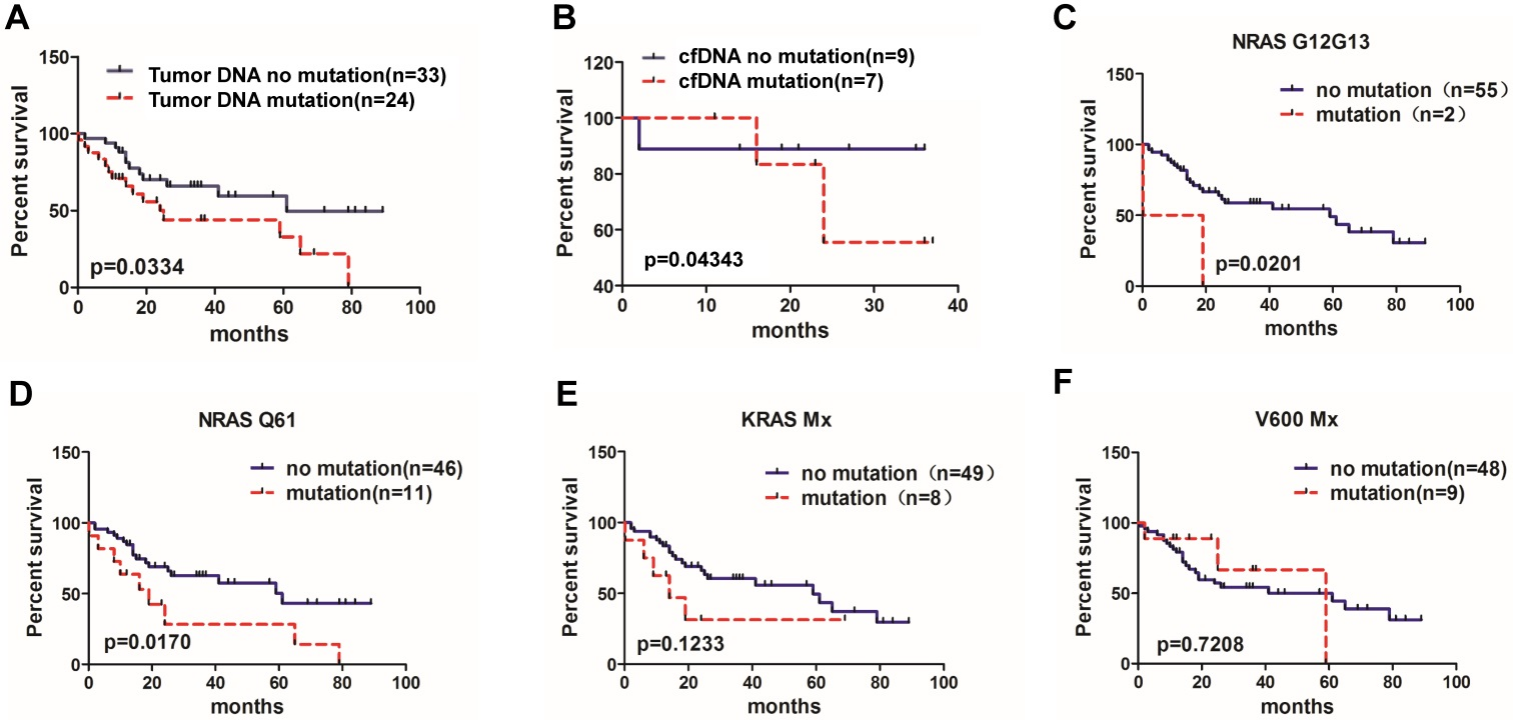
** Correlation of *BRAF*, *KRAS* and *NRAS* mutations and MM patient overall survival rate. (A)** Patients with available data for overall survival analysis according to the bone marrow tumor DNA sample detection (95% CI of rate, 1.972 to 2.908).**(B)** Overall survival analysis according to the cfDNA sample detection (95% CI of rate, 0.04124 to 3.935).** (C)** Overall survival of MM patients with *NRAS* G12/G13 mutation (95% CI of rate, 5.909 to 6.383), **(D)** with *NRAS* Q61 mutation (95% CI of rate, 2.761 to 3.660), **(E)** with *KRAS* Mx mutation (95% CI of rate, 3.836 to 4.593) and **(F)** with V600 Mx mutation (95% CI of rate, 0.7328 to 1.335).

To determine whether *BRAF*, *KRAS* and *NRAS* mutations were associated with patients' outcomes, we analyzed association between overall survival and *BRAF*, *KRAS* and *NRAS* mutation status in 57 patients who had available follow up data including the well-recognized MM prognostic biomarkers, such as serum creatinine, hemoglobin, serum calcium and the ISS stage. As shown in the **Figure 1**, patients with high creatinine level (>2.0 mg/dl) had shorter median survival than those with lower creatinine level (<2.0 mg/dL) (*P*=0.0192) (**Figure 1A**); patients with low hemoglobin level (<85 g/L) had a trend towards shorter median survival (*P*=0.1023) (**Figure 1B**). Also, patients with higher Ca^2+^ level than normal had a trend towards shorter median survival (*P*=0.0745) (**Figure 1C**). In addition, MM patients with ISS stage I and stage II had similar median survival rates (41 months vs. 59 months, *P*=0.661) (**Figure 1D**), but patients at stage III had shorter median survival compared to patients with stages I and II combined together (26 months vs. 59 months, *P*=0.0438) (**Figure 1E**). Finally, comparing tumor DNA results, 24 patients with *BRAF*, *KRAS* and *NRAS* mutations had shorter survival than 33 patients without these mutations (25 months vs. 61months,* P*=0.0334) (**Figure 2A**). At the same time, all 17 patients possessing cfDNA samples were divided into the mutation and no mutation groups accordingly, and the survival analysis also indicated that patients with *BRAF*, *KRAS* and *NRAS* mutations in cfDNA had a shorter median survival than those without mutations (25 months vs. 61 months,* P*=0.04343) (**Figure 2B**).

Subgroup analysis of tumor DNA results showed that the median survival of patients with *NRAS G12/G13* mutations was shorter than that of patients without *NRAS G12/G13* mutations (9.6 months vs. 59 months, *P*=0.0201) (**Figure 2C**). Patients with *NRAS Q61* mutation subgroup demonstrated a significantly shorter median OS compared to patients without *NRAS Q61* (19 months vs. 61 months, *P*=0.0170) (**Figure 2D**). The differences of OS between *KRAS Mx* and *BRAF V600Mx* mutation and non- mutation subgroups had no significant differences (14 months vs 59 months, *P*=0.1233; 59 months vs. 61months, *P*=0.7208, respectively)(**Figure 2E, 2F**).

**Figure 3:**
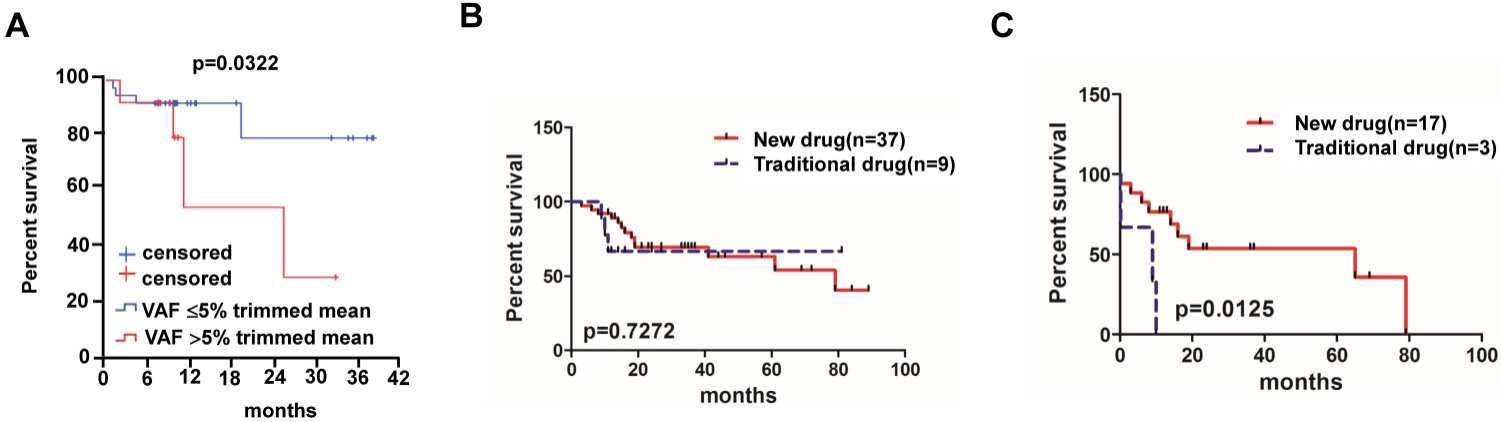
** Overall survival of MM patients treated with traditional chemotherapy or new chemotherapy regimens. (A)** Relationship between VAF rate and survival rate in MM patients (95% CI of rate, 0.00124 to 3.935). **(B)** Different chemotherapy regime and survival rate in MM patients without mutations (95% CI of rate, 0.2013 to 3.060) and **(C)** patients with *BRAF*, *KRAS* and/or *NRAS* mutations (95% CI of rate, 6.952 to 7.493).

Lastly, we analyzed survival VAF for tested mutations in cfDNA and found that patients with high VAF (>5% trimmed mean value) had a shorter median survival than patients with low mutation VAF (≥5% trimmed mean value) (23.8 months vs. not reached, *P*=0.0322;95% CI of rate, 0.04124 to 3.935) (**Figure 3A**).

Of all the 83 MM patients enrolled in our study, 46 patients were administered at least one cycle of chemotherapy. We divided these 46 patients into new drug group (n=37) and traditional drug group (n=9) based on the therapy regimens, where the new drug group means that therapy regimen includes drugs like bortezomib, thalidomide and lenalidomide; otherwise patients were classified in the traditional drug group (i.e. melphalan-dexamethasone regimen, Table 1). Patients receiving new drug therapy regimens (bortezomib-based regimens, Table 1) showed no significant median OS compared to patients treated with traditional drugs (79 months vs. not reached, *P*=0.7272) (**Figure 3B**). Of all the 46 patients, 20 of them with detectable *BRAF*, *KRAS* and/or *NRAS* mutations were also divided into new drug group (n=17) and traditional drug group (n=3) according to the therapy regimens, and patients who had received new agent treatment had a better survival compared to the traditional therapies (65 months vs. 9 months, *P*=0.0125) (**Figure 3C**).

## Discussion

Previous studies demonstrated that *BRAF*, *KRAS* and *NRAS* gene mutations were the main somatic mutations in newly-diagnosed MM 4, 6, 18, 19. In this study, we used ddPCR to detect *BRAF*, *KRAS* and *NRAS* mutations (*BRAF 600Mx, KRAS Mx, NRAS G12/G13, NRAS Q61*) in 83 diagnosed MM patients in both the tumor DNA and for 17 of these patients, also in plasma cfDNA, which is the first study revealing the prevalence and clinicopathological significances of *BRAF*, *KRAS* and *NRAS* mutations in Chinese patients with myeloma using this ddPCR method.

Recently, many studies have shown the value of NGS in the detection of *BRAF*, *KRAS* and *NRAS* mutations in MM 18, 20. The percentage of mutation for *BRAF* is around 4-12%, for *KRAS* is about 20%-30% and for* NRAS* is about 25% 21, 22. Although NGS ensures a higher rate of accuracy identification in MM, uniform and broadly applicable NGS detection protocols are still not available in many academic laboratories. Moreover, NGS has a substantial intrinsic complexity and involves major costs that many institutes and patients might not be able to afford 23. Therefore, PCR based approaches will remain extensively promising in the future.

Compared with NGS, ddPCR is much faster and cheaper. In the present droplet digital PCR system used, the sample reaction mixture is divided into 2×10^4^ droplets per well, and PCR is performed for the target gene(s) of interest. The positively fluorescent droplets contain the target gene allele(s) and those that do not count as negative droplets. Since ddPCR directly counts the positive and negative droplets for a given target which can then be used to directly calculate the target's concentration in a sample well, it offers the advantage of enabling direct and absolute quantitation without requiring comparison to a standard reference curve 24. Some institutes have used this method to detect somatic mutations including *KRAS, XPO1, STAT6* and so on in some solid tumors and lymphomas. Recently, Rustad *et al* reported the first application of ddPCR in detecting mutations of mitogen activated protein kinase pathway genes, and found circulating tumor DNA could reflect MM cell mutation, tumor mass and transformation 14. Drandi *et al.* reported that ddPCR of immunoglobulin gene rearrangement had greater applicability, sensitivity and reduced labor intensiveness than qPCR when using bone marrow and peripheral blood of 18 MM, 21 mantle cell lymphomas and 30 follicular lymphomas 23. In addition, ddPCR and NGS methods have been verified to have high concordance to tumor genotype 17, 25, 26.

In our study, the positive *BRAF*, *KRAS* and *NRAS* mutations in the bone marrow tumor DNA samples were 28/83 (34%), but it was 9/17 (53%) in the plasma cfDNA samples, which demonstrated a significantly higher detection rate. In bone marrow tumor DNA samples, the detection rate of *BRAF V600Mx* mutations was 4/83 (5%), *KRAS Mx* was 13/83 (16%), *NRAS G12/G13* was 3/83 (4%) and *NRAS Q61* was 14/83 (17%), which were approximately equivalent to those found using an NGS method. In 5/17 (29%) cases, we observed the coincident *BRAF*, *KRAS* and *NRAS* mutations in cfDNA matched bone marrow tumor DNA. For further detail analysis, the agreement of cfDNA and tumor DNA of *BRAF V600Mx* was 76%, *KRAS Mx* and *NRAS Q61* mutations were 100%, *NRAS G12/G13* mutations was 100%. Our concordance results for *BRAF*, *KRAS* and *NRAS* mutations compare favorably to most of these published studies despite the fact that we only used a very low amount of cfDNA 19, 21, 27. While bone marrow aspiration is currently required for the diagnosis of MM, it has significant limitations because of either patients' discomfort or the variability of myeloma distribution within the marrow. Nevertheless, BRAF mutation was detected in 4 cfDNA samples but not in their matched tumor samples, to exclude false positive possibility in the analysis of cfDNA samples, it is better to perform DNA sequencing on these 4 samples for BRAF mutation. This limitation of our research should be mindful to the others. Despite the limited number and the discrepancy in special cases of our cohort, it shows encouraging agreement between these two kinds of samples. Hence, detection of plasma cfDNA might an alternative way to bone marrow aspiration.

To further study the prognostic significance of *BRAF*, *KRAS* and *NRAS* mutations, we analyzed the 57 cases which had complete follow-up data. In our study, 24/57 (42%) patients were detected with at least one mutation, and patients with *BRAF*, *KRAS* and *NRAS* mutations had a significantly shorter survival than those without these mutations. Andrulis M *et al.* found that MM patients with *BRAF V600E* mutation tested by mutation-specific immunohistochemistry had a significantly higher incidence of extramedullary disease and a shorter OS 28. Another study enrolling 205 Chinese MM patients showed that *BRAF V600E* mutation presented a poor survival in patients less than 65 years of age 27. Xu *et al* reported that enrichment of *BRAF*, *KRAS* and *NRAS* mutations in relapsed/refractory MM patients was related to a poor survival 22. Our study also provided results consistent with these previous studies.

In our cohort, 46 patients were treated with at least 1 cycle of anti-myeloma treatment and 20 of them had *BRAF*, *KRAS* and *NRAS* mutations. In the current study, we firstly observed that patients with any of these mutations who received new agent treatment had a better survival than those who received traditional drugs. This result suggests that new drugs such as immunomodulatory drugs and proteasome inhibitors might reverse the disadvantage of these gene mutations. But this question still needs to be further investigated using a more comprehensive genetic profiling of MM tumor DNA and cfDNA in serial samples from a larger patient cohort.

## Conclusion

In summary, the molecular analysis of a small amount of unamplified cfDNA for *BRAF*, *KRAS* and *NRAS* mutations in MM patients is feasible and has good concordance with standard mutations testing of bone marrow tumor DNA samples. Our results also suggest that *BRAF*, *KRAS* and *NRAS* mutations might be a prognostic biomarker for OS. But our study still has some potential limitations. We only detected *BRAF V600Mx, KRAS Mx, NRAS G12/G13* and* NRAS Q61* mutations, which can't reflect all mutations of the *BRAF, KRAS* and *NRAS* genes in MM. Furthermore, our clinical data was retrospective and some patients' follow-up information was not available, so the prognostic significance and therapeutic utility still need to be further investigated in future prospective studies.

## Figures and Tables

**Table 1:** Characteristics of patients enrolled in this study.

Characteristic		No. of patients (n=83)
**Age (media in years)**		63(40~83)
**Gender**		
Male		54(65%)
Female		29(35%)
**Stage**		
I		12(14%)
II		23(28%)
III		33(40%)
Not tested		15(18%)
**Type**		
IgG		34(41%)
IgA		12(14%)
IgD		4(5%)
Light chain		19(23%)
Non-secretory		3(4%)
Not test		11(13%)
**Treated patients**		46
Traditional drugs		
	MP or VAD	9 (20%)
New drugs		
	BD or RD	7 (15%)
	BCD or TCD	16(35%)
	PAD	14(30%)

Regimens: MP, melphalan-dexamethasone; VAD, vincristine-doxorubicin- dexamethasone; BD, bortezomib-dexamethasone; RD, lenalidomide-dexamethasone; BCD, bortezomib-cyclophosphamide- dexamethasone; TCD: thalidomide- cyclophosphamide- dexamethasone PAD, bortezomib-doxorubicin-dexamethasone.

**Table 2:** RAS/RAF mutations in different samples.

Mutation test	No. of patients (detectable rate)
**Bone marrow tumor DNA**	83
*BRAF V600Mx*	4 (5%)
*KRAS Mx*	13 (16%)
*NRAS G12/G13*	3 (4%)
*NRAS Q61*	14 (17%)
**Plasma cfDNA**	17
*BRAF V600Mx*	5 (30%)
*KRAS Mx*	1 (6%)
*NRAS G12/G13*	0
*NRAS Q61*	3 (18%)

Note: +:positive; -:negative.

**Table 3:** Agreements for RAS/RAF mutations (tumor DNA vs cfDNA).

patients	BRAFV600Mx			KRAS Mx			NRAS G12/13			NRAS Q61	
	tumor DNA	cf DNA		Tumor DNA	cfDNA		Tumor DNA	cfDNA		Tumor DNA	cfDNA
**MM1**	-	+		-	-		-	-		-	-
**MM2**	-	-		-	-		-	-		+	+
**MM3**	-	-		-	-		-	-		-	-
**MM4**	+	+		-	-		-	-		-	-
**MM5**	-	-		-	-		-	-		+	+
**MM6**	-	+		-	-		-	-		-	-
**MM7**	-	-		+	+		-	-		-	-
**MM8**	-	-		-	-		-	-		-	-
**MM9**	-	-		-	-		-	-		-	-
**MM10**	-	-		-	-		-	-		-	-
**MM11**	-	-		-	-		-	-		-	-
**MM12**	-	-		-	-		-	-		-	-
**MM13**	-	-		-	-		-	-		-	-
**MM14**	-	+		-	-		-	-		-	-
**MM15**	-	-		-	-		-	-		-	-
**MM16**	-	-		-	-		-	-		-	-
**MM17**	-	+		-	-		-	-		+	+
**agreement**	76%			100%			100%			100%	
